# Oligocentric Castleman Disease (OligoCD): A Novel Diagnostic Entity in the Spectrum of Castleman Disease

**DOI:** 10.7759/cureus.100311

**Published:** 2025-12-29

**Authors:** Ritasman Baisya, Juwain Nehil, Vikas Dagar, Sukdev Manna

**Affiliations:** 1 Rheumatology, All India Institute of Medical Sciences (AIIMS) Kalyani, Kalyani, IND; 2 Pathology and Laboratory Medicine, All India Institute of Medical Sciences (AIIMS) Kalyani, Kalyani, IND

**Keywords:** hyaline-vascular variant, imcd, lymphadenopathy, oligocentric castleman disease, rituximab, ucd

## Abstract

Castleman disease (CD) is a rare lymphoproliferative disorder traditionally classified as unicentric (UCD) or multicentric (MCD). However, some cases exhibit features that do not fit either category. These atypical cases warrant recognition as a distinct subtype, proposed here as oligocentric Castleman disease (OligoCD).

A 38-year-old woman presented with bilateral lacrimal gland swelling and cervical lymphadenopathy, fatigue, and low-grade fever. Laboratory investigations, including hemogram, liver and kidney function tests, inflammatory markers, and interleukin-6 (IL-6), were within normal limits, except for mildly elevated erythrocyte sedimentation rate (ESR) and immunoglobulin G4 (IgG4) levels, elevated to approximately twice the upper limit of normal. Imaging did not reveal any other lymphadenopathy or systemic involvement. Lymph node biopsy revealed features of the hyaline-vascular variant of Castleman disease, confirmed on immunohistochemistry. The differential diagnosis of IgG4-related disease (RD) was considered but ruled out due to the non-fulfillment of the clinical diagnostic criteria and the absence of plasma cell infiltrate on histopathological examination. The presentation, however, did not fulfil the criteria for either UCD or MCD. She had an excellent response to prednisolone initially and a complete response to rituximab.

This case underscores the need to broaden the existing diagnostic framework for Castleman disease. The clinical and histopathological findings favor recognizing oligocentric Castleman disease as a novel intermediate subtype within the disease spectrum.

## Introduction

Castleman disease (CD) comprises a rare group of non-clonal lymphoproliferative disorders, subclassified into unicentric (UCD) and multicentric (MCD) variants. UCD typically involves a single lymph node region without systemic symptoms, whereas MCD affects multiple lymph node regions and is associated with systemic inflammation, often driven by elevated interleukin-6 (IL-6) levels or human herpesvirus-8 (HHV-8) infection [1,2].

However, many cases cannot be clearly categorized into either UCD or MCD. Some patients present with mild inflammatory symptoms despite normal inflammatory markers, while others show the enlargement of more than one lymph node but lack systemic inflammatory features. These presentations suggest that CD represents a spectrum of disease rather than a strictly biphasic entity.

For such cases, which do not fulfil the diagnostic criteria for either UCD or idiopathic MCD (iMCD), a new intermediate term has recently been proposed by the ACCELERATE study group [3]. These observations call for the re-examination of the current classification system. Here, we present such a case and support the term oligocentric Castleman disease (OligoCD) to describe this hybrid presentation.

## Case presentation

**Figure 1:**
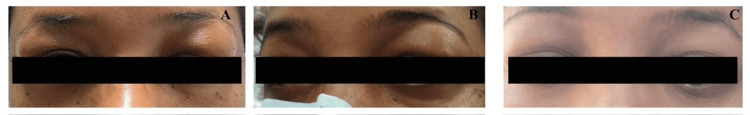
Progression of supraorbital swelling in the patient (A) Supraorbital swelling on the first visit. (B) After three months, the reappearance of supraorbital swelling after tapering prednisolone to <15 mg/day. (C) Supraorbital swelling after two doses of rituximab 15 days apart

A 38-year-old woman presented with bilateral upper eyelid swelling lasting one year, with rapidly increasing swelling in the neck over the past three months. She also reported abdominal pain, episodes of chronic diarrhea, low-grade fever, and persistent fatigue. Her medical history included uncontrolled diabetes mellitus and hypothyroidism. Clinical examination revealed bilateral upper eyelid swelling (left > right) and bilateral cervical lymphadenopathy (left > right). Figure 1 shows the clinical progression of the patient with supraorbital swelling. At the first visit, a prominent supraorbital swelling is seen (Figure 1A). After three months of corticosteroid therapy, the swelling reappeared upon tapering prednisolone below 15 mg/day (Figure 1B). Figure 1C shows the complete resolution of the supraorbital swelling following two doses of rituximab, indicating a favorable therapeutic response. There were no other lymphadenopathies or evidence of hepatosplenomegaly.

**Figure 2:**
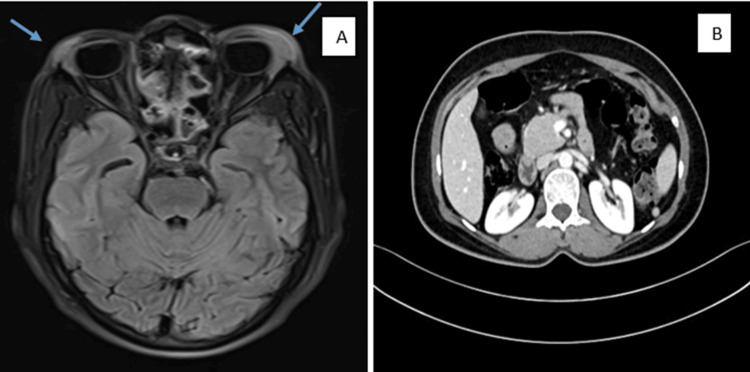
Radiological investigation (A) T2-FLAIR image of the brain, including the orbit, showing hyperintensity in both lacrimal glands (left more than right). (B) CECT of the whole abdomen showing no significant lymphadenopathy FLAIR, fluid-attenuated inversion recovery; CECT, contrast-enhanced CT

Investigations showed a normal hemogram, with a mildly elevated erythrocyte sedimentation rate (ESR) and an increased glycated hemoglobin (HbA1c) level. Liver and renal function tests were within normal limits. Viral serologies, including HIV and HHV-8, were negative. Immunological evaluation revealed elevated immunoglobulin G4 (IgG4) levels, with normal IL-6, as shown in Table 1. An antinuclear antibody (ANA) qualitative blot panel was performed, assessing antibodies to double-stranded deoxyribonucleic acid (dsDNA), nucleosome, histone, SmD1, PCNA, PO, SS-A/Ro 60 kD, SS-A/Ro 52 kD, SS-B/La, CENP-B, Scl-70, U1-snRNP, AMA-M2, Jo-1, PM-Scl, Mi-2, Ku, and DFS70; all were negative. The MRI of the brain with orbit demonstrated the bilateral enlargement of the lacrimal glands, with homogeneous contrast enhancement and diffuse restriction, as shown in Figure 2A. The contrast-enhanced CT (CECT) of the abdomen showed mild pancreatic swelling. However, magnetic resonance cholangiopancreatography (MRCP) ruled out any definite evidence of pancreatitis, supported by normal amylase and lipase levels. There was no evidence of mediastinal, hilar, or abdominal lymphadenopathy. The differential diagnosis of IgG4-related disease (RD) was considered; however, according to the 2019 American College of Rheumatology (ACR)-European League Against Rheumatism (EULAR) criteria, although entry criteria were met (bilateral lacrimal gland involvement), exclusion criteria were present (Castleman disease, hyaline-vascular variant on histopathology), and the inclusion score was 12 (<20); thus, IgG4-RD was ruled out, with no plasma cells seen on biopsy, so IgG4 immunostaining was not performed.

**Table 1:** Laboratory investigation report with reference ranges ESR, erythrocyte sedimentation rate; CRP, C-reactive protein; anti-CCP, anti-cyclic citrullinated peptide; HbA1c, glycated hemoglobin; TSH, thyroid-stimulating hormone; T3, triiodothyronine; T4, thyroxine; SGOT/AST, serum glutamic oxaloacetic transaminase/aspartate aminotransferase; SGPT/ALT, serum glutamic pyruvic transaminase/alanine aminotransferase; PT/aPTT, prothrombin time/activated partial thromboplastin time; ANA, antinuclear antibody; IgG4, immunoglobulin G4

Serial no	Investigation	Report	Reference range
1	Hemoglobin	15.4 g/dL	12-15 g/dL
2	Total leukocyte count	11.99 × 10^3^/μL	4-10 × 10^3^/μL
3	Platelet count	323 × 10^3^/μL	150-450 × 10^3^/μL
4	ESR	31 mm/hour	0-20 mm/hour
5	Serum CRP	6.9 mg/L	≤10 mg/L
6	Rheumatoid factor	8.6 IU/mL	<12 IU/mL
7	Anti-CCP	3.55 μ/mL	<20 μ/mL
8	HbA1c	10.2	<6.5, diabetes
9	TSH level	4.25 μIU/mL	0.38-5.33 μIU/mL
10	T3 level	0.77 ng/mL	0.87-1.78 ng/mL
11	T4 level	12.09 μg/dL	6.09-12.23 μg/dL
12	Serum urea	15 mg/dL	7-20 mg/dL
13	Serum creatinine	0.42 mg/dL	0.6-1.1 mg/dL
14	Total bilirubin	1.1 mg/dL	0.1-1.2 mg/dL
15	SGOT/AST	11 U/L	5-40 U/L
16	SGPT/ALT	18 U/L	7-56 U/L
17	Alkaline phosphatase	72 U/L	40-129 U/L
18	PT/aPTT	12.6 seconds/27 seconds	10-13 seconds/25-35 seconds
19	ANA (quantitative)	Negative	Negative
20	Interleukin-6	3.67 pg/mL	0.00-7.00 pg/mL
21	IgG4 levels	>4.78 g/dL	0.03-2.01 g/dL
22	Serum amylase level	51 IU/L	30 to 110 IU/L
23	Serum lipase level	210 U/L	23 to 300 U/L

**Figure 3:**
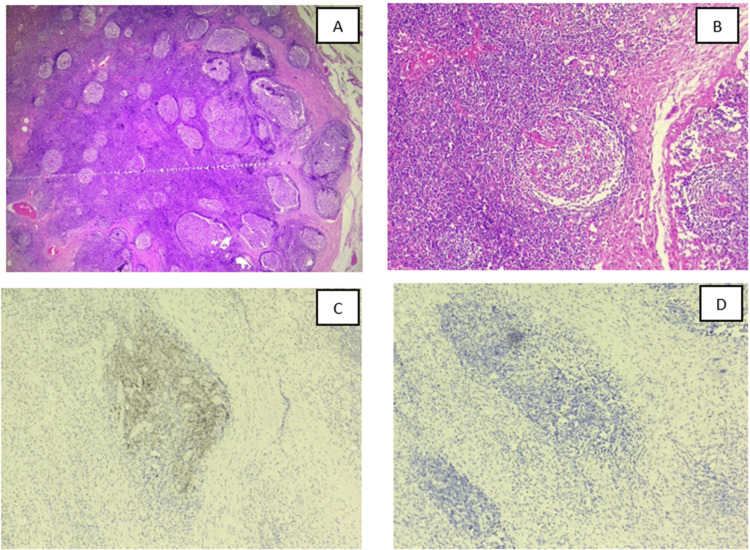
Histopathology of left supraclavicular lymph node biopsy tissue (A) H&E-stained scanner view (4×) from the left supraclavicular lymph node showing that the lymph node architecture is partially effaced by numerous reactive lymphoid follicles of variable size. (B) Many germinal centers are regressed and hyalinized, with penetrating hyalinized capillaries giving a characteristic “lollipop” appearance. (C) Immunohistochemistry for CD21 demonstrates expanded and dysplastic follicular dendritic cell (FDC) meshworks within hyalinized germinal centers, confirming reactive follicular hyperplasia. (D) Immunohistochemistry for HHV-8 negativity, excluding HHV-8-associated multicentric Castleman disease CD21, cluster of differentiation 21; HHV-8, human herpesvirus-8

An excision biopsy from the left supraclavicular lymph node, as shown in Figure 3, revealed hyalinized, atrophic germinal centers with sclerotic blood vessels forming “lollipop” follicles, thickened mantle zones with concentric layering (“onion-skin” appearance), and the proliferation of follicular dendritic cells. Immunohistochemistry showed cluster of differentiation 21 (CD21) positivity, CD23 negativity, and HHV-8 negativity. The biopsy was suggestive of Castleman disease. The lacrimal gland biopsy was planned but was declined by the patient. Based on histology and immunohistochemistry, the diagnosis was Castleman disease, hyaline-vascular variant. However, the patient’s presentation was inconsistent with both UCD (due to systemic symptoms and lacrimal gland involvement) and MCD (due to single-site nodal involvement and normal IL-6). Therefore, the diagnosis was given as oligocentric Castleman disease.

The patient was treated with steroids 0.5 mg/kg, prednisolone (25 mg), with gradual tapering for three months and responded well to prednisolone until 20 mg/day. She reported the recurrence of periorbital swelling when reducing the prednisolone dose below 15 mg/day; hence, she was planned to receive injectable rituximab 1 g in two doses 15 days apart. After initiating rituximab and gradually tapering steroids, her symptoms improved significantly.

## Discussion

Castleman disease poses diagnostic challenges due to its heterogeneous clinical and histological features. UCD is generally asymptomatic and localized, while MCD includes systemic features such as fever, anemia, and multiorgan involvement. The hyaline-vascular variant of CD, typically associated with UCD, is defined by small, regressed germinal centers; prominent vasculature; and follicular dendritic cell expansion [4,5].

In our case, the histological findings were classic for hyaline-vascular CD, yet the systemic symptoms did not define either UCD or MCD [1,2,6]. The ACCELERATE registry study, led by Pierson et al., reported that approximately 7.8% of all Castleman disease cases represent diagnostically ambiguous, intermediate presentations and emphasized the need for more refined classification [3,7]. The authors proposed oligocentric Castleman disease (OligoCD) to describe these cases, characterized by solitary or oligo-regional lymph node involvement, mild non-disabling systemic symptoms, normal or minimally elevated IL-6 levels, HHV-8 negativity, and classic histopathological features of Castleman disease.

The recognition of OligoCD may prevent the overtreatment and misclassification of patients with atypical CD. The introduction of OligoCD also aligns with the need for nuanced classifications in rare lymphoproliferative disorders. As in literature, we are finding many such atypical CD cases; we must consider that this disease has a spectrum of symptoms rather than being limited to UCD or MCD only.

Lacrimal gland involvement in CD is rare and can present as an orbital lymphoproliferative disorder with nonspecific clinical and imaging features [8]. Case reports and small series describe patients with mass lesions in the lacrimal gland area, usually causing eyelid swelling, ptosis, and other space-occupying symptoms [9]. However, histopathology is needed to exclude malignant lymphoproliferation, which was not performed in our case. Given that the patient exhibited features of CD in the cervical lymph node and responded well to immunosuppressive therapy, it was hypothesized that the lacrimal gland involvement was a manifestation of CD in this case.

In comparison to various subtypes of Castleman disease, as shown in Table 2, unicentric Castleman disease (UCD) typically involves a single lymph node and is generally localized, whereas multicentric Castleman disease (MCD) presents with multiple nodal enlargements involving several regions. The oligoclonal or oligocentric variant (OligoCD) shows the involvement of single or a few regional nodes, representing an intermediate pattern between UCD and MCD [7,10].

**Table 2:** Comparison of CD subtypes in various studies IL-6, interleukin-6; HHV-8, human herpesvirus-8; IgG4, immunoglobulin G4; UCD, unicentric Castleman disease; MCD, multicentric Castleman disease; OligoCD, oligocentric Castleman disease; CD, Castleman disease

Feature	UCD	MCD	OligoCD
Nodal involvement[7,10]	Single	Multiple	Single or oligo-regional
Systemic symptoms [2,7,11]	Absent	Present	Mild fatigue/fever
IL-6 [11,12]	Normal	Elevated	Normal or mildly elevated
Histopathology [7,12]	Hyaline vascular (most common)	Plasma cell or mixed type predominates	Hyaline vascular pattern with/without plasma cell-rich areas
HHV-8 [2,7]	Negative	Often positive	Negative
Overlap with IgG4-related disease [7,11]	Occasional overlap	Overlap possible	Few reported overlaps mimicking IgG4-related disease
Radiological findings [7,13]	Solitary, well-defined mass	Generalized lymphadenopathy	Multiple contiguous nodal enlargements
Treatment [14,15]	Surgery	Systemic therapy	Surgery ± monitoring, often radiation/chemotherapy
Prognosis [7,10,13]	Excellent	Variable	Favorable (under-studied)

Systemic symptoms are usually absent in UCD, while patients with MCD frequently exhibit constitutional manifestations such as fever, malaise, and weight loss. In contrast, OligoCD may present with only mild fatigue or low-grade fever. In terms of viral association, HHV-8 infection is typically absent in UCD, frequently present in MCD (especially in HIV-positive cases), and negative in OligoCD. Occasional overlap with IgG4-related disease has been described in UCD, while such overlap is also possible in MCD, and a few rare reports exist for OligoCD that mimic IgG4-related lymphadenopathy [2,7,11].

Serum interleukin-6 (IL-6) levels are normal in UCD, elevated in MCD, and normal to mildly increased in OligoCD. Histopathologically, UCD most often exhibits the hyaline vascular type, MCD usually demonstrates a plasma cell or mixed variant, and OligoCD may show hyaline vascular morphology with or without plasma cell-rich areas [7,12].

Radiologically, UCD presents as a solitary, well-defined enhancing mass, while MCD shows generalized lymphadenopathy with hepatosplenomegaly or effusions. OligoCD typically demonstrates multiple contiguous or regional nodal enlargements, sometimes with moderate enhancement [13].

Treatment strategies vary according to disease extent. Surgical excision is curative in UCD, whereas MCD requires systemic therapy, including anti-IL-6 agents, corticosteroids, or antivirals [14,15]. OligoCD may respond to surgical excision with or without adjuvant radiotherapy or chemotherapy, and close monitoring is often advised. The prognosis is excellent in UCD, variable in MCD depending on subtype and response, and generally favorable in OligoCD, though this category remains under-studied [10,13].

## Conclusions

This case underscores the evolving understanding of Castleman disease and the need for refinement beyond the traditional unicentric-multicentric (UCD-MCD) framework. Although histopathologically consistent with UCD, the patient demonstrated systemic features not fulfilling MCD criteria. This case reinforces the recognition of oligocentric Castleman disease (OligoCD) as a distinct, intermediate subtype with unique diagnostic and therapeutic implications. Embracing this terminology can enhance diagnostic precision, promote individualized treatment, and help prevent overtreatment, undertreatment, and misdiagnosis while reducing the misclassification of overlapping entities such as IgG4-related disease, lymphoproliferative disorders, and Castleman-like disease.
